# Spin Waves and Magnetic Exchange Hamiltonian in CrSBr

**DOI:** 10.1002/advs.202202467

**Published:** 2022-07-07

**Authors:** Allen Scheie, Michael Ziebel, Daniel G. Chica, Youn June Bae, Xiaoping Wang, Alexander I. Kolesnikov, Xiaoyang Zhu, Xavier Roy

**Affiliations:** ^1^ Neutron Scattering Division Oak Ridge National Laboratory Oak Ridge TN 37831 USA; ^2^ Department of Chemistry Columbia University New York NY 10027 USA

**Keywords:** magnetism, neutron scattering, spin waves, 2D materials

## Abstract

CrSBr is an air‐stable two‐dimensional (2D) van der Waals semiconducting magnet with great technological promise, but its atomic‐scale magnetic interactions—crucial information for high‐frequency switching—are poorly understood. An experimental study is presented to determine the CrSBr magnetic exchange Hamiltonian and bulk magnon spectrum. The *A*‐type antiferromagnetic order using single crystal neutron diffraction is confirmed here. The magnon dispersions are also measured using inelastic neutron scattering and rigorously fit the excitation modes to a spin wave model. The magnon spectrum is well described by an intra‐plane ferromagnetic Heisenberg exchange model with seven nearest in‐plane exchanges. This fitted exchange Hamiltonian enables theoretical predictions of CrSBr behavior: as one example, the fitted Hamiltonian is used to predict the presence of chiral magnon edge modes with a spin‐orbit enhanced CrSBr heterostructure.

## Graphical abstract

CrSBr is a exfoliatable two‐dimensional (2D) van der Waals magnet which becomes ferromagnetic in the monolayer limit. The spin wave spectrum measured by neutron scattering, and the magnetic Hamiltonian fitted using linear spin wave theory is reported. The results give an accurate model which will allow detailed simulation of the high‐frequency and topological magnetic behavior of the system.

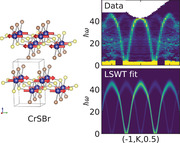

## 1 Introduction

Two‐dimensional (2D) magnetism has long been a topic of theoretical investigation, but only recently has it become experimentally accessible through van der Waals materials.^[^ ^1^, ^2^ ^]^ With monolayer magnetism preserved through magnetic anisotropy, these materials promise to yield cleaner experimental realizations of theoretical states, novel spintronic devices, and new topological phases of matter.^[^ ^3^, ^4^ ^]^ However, accurately predicting the magnetic properties and excitations requires a detailed knowledge of the magnetic exchange Hamiltonian.

**Figure 1:**
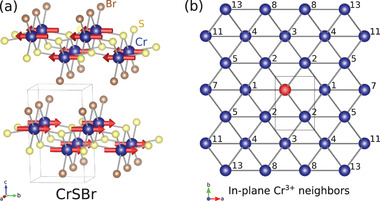
CrSBr crystal structure. a) The crystal structure and Cr^3 +^ magnetic order, ferromagnetic in‐plane but layered in alternating directions for a bulk antiferromagnetism is shown. b) The Cr neighbors in the plane from the central red atom, numbered in order of bond length (neighbors 6, 9, 10, and 12 are between planes) are shown.

A promising 2D van der Waals magnet is CrSBr. CrSBr forms in 2D layers of magnetic Cr^3 +^ ions forming a rectangular lattice, as shown in **Figure** **1**. In bulk, it orders magnetically at *T*_N_ = 132 K^[^ ^5^, ^6^, ^7^ ^]^ with A‐type antiferromagnetism: ferromagnetic planes polarized along the *b*‐axis, alternating in orientation for an overall antiferromagnetic (AFM) order, and becoming ferromagnetic (FM) in the monolayer limit.^[^ ^8^ ^]^ This material is air‐stable and has a semiconducting gap low enough to gate with realistic electric fields.^[^ ^6^, ^9^ ^]^ There is also intricate interplay between electronic transport, optical properties, and magnetism,^[^ ^10^, ^11^ ^]^ with the potential for exploiting both spin and charge degrees of freedom for technological purposes.^[^ ^12^ ^]^ To understand, predict, and ultimately exploit the spin transport properties of CrSBr, it is necessary to know the magnetic exchange Hamiltonian between Cr ions and the resulting magnon dispersions. In particular, the high‐frequency behavior of the magnon bands is critical to understanding the short‐time behavior relevant for electronic switching and information processing. In this study, we measure the static magnetic structure and the high energy magnon dispersions, experimentally determine the spin exchange Hamiltonian using inelastic neutron scattering, and then use this Hamiltonian to predict the presence of chiral edge modes in layered heterostructures. In this way, using bulk probes yields crucial information to predict monolayer behavior.

## 2 Results and Analysis

### 2.1 Static Magnetism

**Figure 2:**
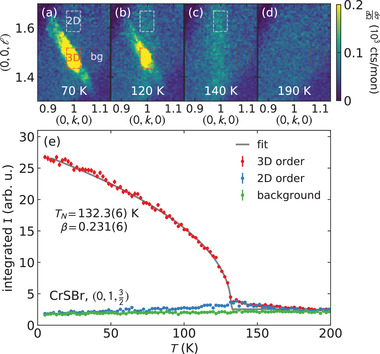
Single crystal CrSBr neutron diffraction. a– d) The temperature evolution of the $\left(0,1,\frac{3}{2}\right)$ Bragg peak (which is smeared out in *Q*_⊥_ due to crystal twinning) is shown. Above and near the phase transition, a streak of scattering along ℓ appears, signaling two‐dimensional (2D) magnetic correlations. We track the 3D and 2D correlations using the red and white boxes in panels (a– d) (the black box is the background), plotted in panel (e). The 2D correlations peak at the ordering temperature *T*_N_ = 132.2(6) K, and decrease at lower temperatures. The fitted order parameter curve is shown in gray, with a fitted β = 0.231(6). Error bars indicate one standard deviation uncertainty.

The single crystal neutron diffraction is shown in **Figure** **2** and confirms the ground state magnetic order in ref. [7]: below a transition temperature of 132.3(6) K, new Bragg peaks appear at half‐integer ℓ positions in accord with $\left(00\frac{1}{2}\right)$ magnetic order. In the Supporting Information, we refine the Bragg intensities and show they indicate A‐type antiferromagnetism in Figure 1. At temperatures near *T*_N_, a streak of scattering appears at (0, 1, ℓ), signaling 2D magnetic correlations in the *ab*‐plane. Tracking the 2D correlations as a function of temperature, we see they peak at *T*_N_, but with significant 2D magnetic correlations above *T*_N_.

The 3D Bragg intensity versus temperature follows a smooth curve between *T*_N_ and 5 K. Although CrSBr samples show a sample‐dependent discontinuity in magnetic susceptibility at 30 K,^[^ ^10^ ^]^ no such feature is observed in the neutron diffraction. Furthermore, aside from a larger static moment at 5 K, we also find no difference between 80 and 5 K refined magnetic structures. Thus we conclude, as did ref. [7], that the 30 K discontinuity is not associated with a change in the spatially‐averaged magnetic order. This is consistent with the proposal in ref. [10] that the susceptibility discontinuity is due to local or impurity spins. Fitting the 3D Bragg intensity to an order parameter curve, we find a critical exponent β = 0.231 ± 0.006. This is far from the theoretical 3D Heisenberg critical exponent β = 0.36.^[^ ^13^ ^]^ Instead, this is remarkably close to the critical exponent β = 0.231 derived for the 2D *XY* model via Kosterlitz– Thouless (K– T) theory,^[^ ^14^ ^]^ showing very 2D exchange interactions with easy‐plane anisotropy, in accord with expected Van der Waals behavior.

### 2.2 Dynamic Magnetism

**Figure 3:**
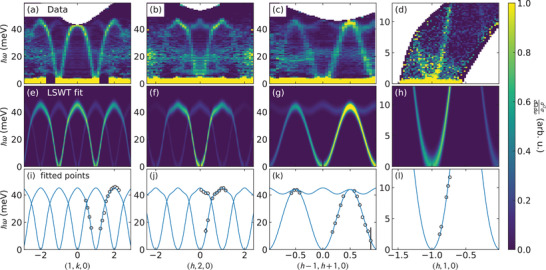
Measured and fitted spin wave spectra of CrSBr. The top row (a– d) shows the measured spin wave spectra of CrSBr. Panels (a– c) were measured with *E*_*i*_ = 70 meV neutrons, while panel (d) was measured with *E*_*i*_ = 20 meV neutrons. The middle row (e– h) shows the LSWT calculated spectrum from the best fit Hamiltonian in Table 1. The bottom row (i– l) shows a portion of the data points used in the fit (black circles), and the fitted dispersion (blue solid line). For a complete list of fitted data, see the Supporting Information.

Several plots of CrSBr inelastic neutron scattering data are shown in **Figure** **3**. Because of the small sample mass, there is substantial background noise from phonon scattering in the aluminum sample holder. Nevertheless, the magnon modes are clearly distinguished by i) their symmetries following the CrSBr reciprocal lattice units, ii) their intensities following a magnetic form factor with intensity largest near |*Q*| = 0, and iii) comparison with a measured background (see Experimental Section).

The magnon dispersions in the (*hk*0) plane reach a maximum energy of ≈45 meV. Within an energy resolution of ±0.5 meV FWHM in the *E_i_* = 20 meV data (Figure 3d), the modes are gapless at *h* + *k* = even integer points in reciprocal lattice units (RLU). This lack of observable gap evidences highly isotropic magnetism, as one expects for *S* = 3/2 Cr^3 +^. This comports with density functional theory predictions^[^ ^15^ ^]^ and recent photon measurements finding *Q* = 0 magnon gaps of 0.102(3) and 0.141(4) meV,^[^ ^16^ ^]^ as well as magnetization measurements finding a maximum anisotropy of 0.144 μeV at 2 K (*c*‐axis compared to *b*) in CrSBr (see Supporting Information^[^ ^17^ ^]^): too small to be resolved in this experiment.

**Figure 4:**
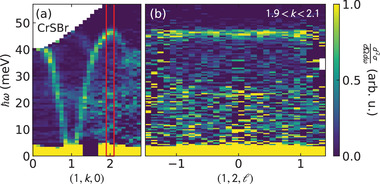
CrSBr dispersion along ℓ. Panel (a) shows a cut along (1, *k*, 0) with red lines delineating the cut in panel (b) along (1, 2, ℓ). There is no detectable dispersion along ℓ at this or any other wavevector, showing that the inter‐plane magnetic exchange is negligibly weak.

In the ℓ‐direction, we find no measurable dispersion out of plane at all *h* and *k*, as shown in **Figure** **4**. This evidences very weak inter‐plane magnetic exchange, as one would expect for a highly 2D system (see the Supporting Information for further details^[^ ^17^ ^]^). This is consistent with the photon excitation study in ref. [16] which finds an interlayer exchange <0.01 meV, as well as density functional calculations in ref. [18] which finds a CrSBr interlayer magnetic interaction three orders of magnitude weaker than the in‐plane interactions. Because the modes are flat with ℓ, all in‐plane scattering data presented here is integrated over −1 < ℓ < 1 RLU to maximize the magnon mode visibility.

### 2.3 Fitting the Exchange Hamiltonian

We determined the CrSBr magnetic exchange constants from this scattering data by performing a fit to a linear spin wave theory (LSWT) model. The spin wave model for a bipartite ferromagnetic lattice is calculated following ref. [19] using the Hamiltonian

(1) $$H=\sum_{i,j}J_{\left\langle ij\right\rangle }\vec{S_{i}}\cdot \vec{S_{j}}$$

where $\vec{S_{i}}$ are vectors of length $|\vec{S_{i}}|=3/2$ and *J*_〈*ij*〉_ are magnetic exchange constants between pairs of spins. Because many exchanges are symmetry‐equivalent, we write *J*_*n*_ where *n* is the neighbor number. The fitted neighbors *n* are shown in Figure 1b.

To constrain the fit, we extracted 188 unique *Q* and ℏω points by fitting constant |*Q*| cuts of the magnon modes to Gaussian profiles in energy across 11 different data slices, using only regions where the magnons are clearly distinguishable from background (see Supporting Information for details^[^ ^17^ ^]^). We then defined a global reduced χ^2^ function based on magnon mode energies at those *Q* points, minimizing $\chi_{\mathrm{red}}^{2}$ by varying *J*_*n*_ using Scipy's optimization package.^[^ ^20^ ^]^

**Figure 5:**
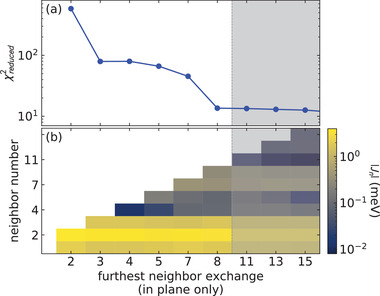
Dependence of the best fit $\chi_{\mathrm{red}}^{2}$ on the number of neighbors included in the fit. Panel (a) shows $\chi_{\mathrm{red}}^{2}$ versus neighbor number *n*, and panel (b) shows these fitted values in a colormap. Beyond the eighth neighbor, the $\chi_{\mathrm{red}}^{2}$ does not appreciably improve by adding additional neighbors, so we truncate our model at the eighth neighbor.

To systematically determine the number of exchange constants to include in our model, we fitted the magnon modes to a spin wave model beginning with only two neighbors, and increasing the number of neighbors up to the 17th neighbor exchange (excluding all inter‐plane exchanges), re‐fitting for each new neighbor. We find that additional neighbors improve the best fit $\chi_{\mathrm{red}}^{2}$ value up to the eighth neighbor (excluding *J*_6_, which is between CrSBr planes). Including neighbors beyond eight does not improve $\chi_{\mathrm{red}}^{2}$ by a significant amount, as shown in **Figure** **5**. Furthermore, we find that the statistical uncertainty of all exchanges beyond the eighth neighbor overlap with zero, and so we truncate our model at the eighth neighbor exchange and consider all further exchanges to be negligible in CrSBr.

The best fit CrSBr Hamiltonian is given in **Table** **1**. Uncertainty was calculated via a $\Delta \chi_{\mathrm{red}}^{2}=1$ contour for a one standard deviation statistical uncertainty,^[^ ^21^ ^]^ see Supporting Information for details.^[^ ^17^ ^]^ This was added in quadrature to the systematic uncertainty from truncating the model to the eighth neighbor exchange, taken to be the range of parameter variation between *n* = 11 and *n* = 17 fits. We simulated the neutron cross section for this best fit Hamiltonian using *SpinW* software package,^[^ ^22^ ^]^ plotted in Figure 3e–h.

**Table 1:** Best fit Hamiltonian exchange parameters for CrSBr. Uncertainty indicates one standard deviation

*J*_1_ =	−1.90 ± 0.10 meV	*J*_5_ =	−0.09 ± 0.06 meV
*J*_2_ =	−3.38 ± 0.06 meV	*J*_7_ =	0.37 ± 0.09 meV
*J*_3_ =	−1.67 ± 0.10 meV	*J*_8_ =	−0.29 ± 0.05 meV
*J*_4_ =	−0.09 ± 0.05 meV		

The agreement between theory and experiment is remarkably good for this isotropic exchange model. However, asymmetric Dzyaloshinskii– Moriya (DM) exchange $H={\vec{D}}_{\left\langle ij\right\rangle }\cdot \left(\vec{S_{i}}\times \vec{S_{j}}\right)$ is symmetry‐allowed on the nearest neighbor Cr−Cr bond, with a ${\vec{D}}_{1}$ vector along the *b*‐direction.^[^ ^23^ ^]^ This DM interaction would produce a magnon mode splitting at half‐integer *k* wavevectors. Although this exchange is expected to be weak in Cr^3 +^ because of its small spin‐orbit coupling, such mode splitting was observed in CrI_3_ with a fitted DM interaction of 0.31 meV.^[^ ^4^ ^]^ Thus it may be that a weak DM exchange also plays a role in CrSBr.

**Figure 6:**
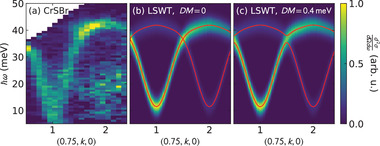
Effect of nearest neighbor DM interaction on the CrSBr dispersion. Panel (a) shows the CrSBr data along (0.75, *k*, 0), and panels (b) and (c) show the LSWT predictions with and without a DM term. The DM induces a gap at *k* = 1.5, but no gap is resolvable in the data. This constrains the nearest neighbor DM term to be <0.8 meV.

To test whether the DM exchange is significant, we added a nearest neighbor DM exchange to our fitted model and allowed it to vary along with the other fitted parameters. No mode splitting is observed in our data, so any split modes are below the experimental resolution (see **Figure** **6**). We find that the best fit nearest neighbor ${\vec{D}}_{1}$ is unstable against the number of neighbors *n* included in the model, varying between 0.0 and 0.4(4) meV. We also find that the uncertainty overlaps with zero for all *n*. Furthermore, the best fit $\chi_{\mathrm{red}}^{2}$ slightly *worsens* when the DM exchange is added: $\chi_{\mathrm{red}}^{2}=13.5819$ with ${\vec{D}}_{1}$, $\chi_{\mathrm{red}}^{2}=13.5818$ without ${\vec{D}}_{1}$ (see Supporting Information^[^ ^17^ ^]^). DM exchange is symmetry‐forbidden on the second neighbor bond, but is allowed on the third neighbor bond where the signature is also mode splitting at the Brillouin zone boundary. No such splitting is resolvable in the data. Therefore, we consider the DM exchange to be negligible for CrSBr. While it is presumably nonzero, it is too small to resolve using this data.

## 3 Discussion

These results show that the CrSBr spin exchange Hamiltonian can be accurately approximated as a single‐layer ferromagnet. Single‐ion anisotropy, inter‐plane exchange, and anisotropic exchange are all too small to resolve, leaving the exchange constants in Table 1 as an effective minimal model for the high frequency (short time) behavior of CrSBr. The fitted exchange parameters are almost uniformly ferromagnetic, with very similar exchange in the *a*‐ and *b*‐directions, evidencing very 2D magnetism (in contrast to the quasi‐1D electronic bands^[^ ^24^ ^]^). The CrSBr magnetic Hamiltonian having significant magnetic exchange out to the eighth neighbor is somewhat surprising, but is consistent with the strong Cr−S and Cr−Br covalency^[^ ^18^ ^]^ which gives opportunity for extended orbital overlap.

We can compare this with first principles predictions for CrSBr. Guo et al.,^[^ ^25^ ^]^ used density functional theory to predict *J*_1_ = −1.72 meV, *J*_2_ = −3.25 meV for CrSBr (normalized to the *S* = 3/2 vector convention we use in Equation (1)). This is very close to the fitted *J*_1_ = −1.9(1) meV and *J*_2_ = −3.38(6) meV, showing good agreement between experiment and theory. Similarly, Wang et al.^[^ ^15^ ^]^ and Yang et al.^[^ ^18^ ^]^ also used density functional theory on to predict weak CrSBr single‐ion anisotropy (too weak to be measured with our measurements), although both their calculated CrSBr bulk exchange constants are larger than we observe in experiment.

Because the CrSBr semiconducting gap is 1.25(7) eV^[^ ^6^ ^]^ (14500 K), the effects of thermally populated conduction‐mediated exchange will be very minor between 5 and 300 K. Some exchange constant shifts with lattice expansion is possible, but such effects will also be minor.^[^ ^26^ ^]^ Therefore we expect the magnetic exchange constants in Table 1 can be considered approximately correct at all temperatures below 300 K.

### 3.1 Calculating Edge Modes

Having determined the spin exchange Hamiltonian for CrSBr, we can begin using it to calculate relevant quantities. Among many spintronics proposals are “topological magnonics”: using magnon edge modes for low‐dissipation transport and switches.^[^ ^27^ ^]^ Magnon edge states, which only exist on the edge of a 2D material, generally have different dispersions than those in the bulk. For certain lattice geometries and Hamiltonians, the edge magnons can be “chiral”, with a directional velocity preference based on the terminating surface.^[^ ^28^ ^]^ Such chiral edge modes can be induced in ferromagnets via an anisotropic DM interaction.^[^ ^29^ ^]^

In 2D materials, it is possible to increase the anisotropy via proximity effects with layers of heavy atoms, thereby enhancing spin‐orbit interaction.^[^ ^30^, ^31^ ^]^ This has been powerfully demonstrated with graphene heterostructures.^[^ ^32^, ^33^ ^]^ Because spin orbit interaction drives the asymmetric DM exchange,^[^ ^23^ ^]^ it is possible to increase the CrSBr DM interaction via layering with a strong spin‐orbit coupled material.^[^ ^34^, ^35^ ^]^

**Figure 7:**
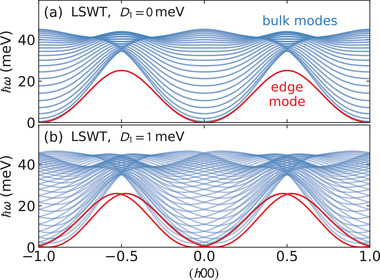
Large box linear spin wave theory (LSWT) simulations of CrSBr with lattice 12 unit cells along *b*. The surface magnon modes are plotted in red, while the bulk magnon modes are plotted in blue. When *D*_1_ becomes nonzero, the surface modes split and have linear crossings at integer and half‐integer *h*, signaling potential chiral edge modes with opposite group velocities on opposing edges.

To examine the effect of large DM exchange on the surface magnon modes of CrSBr, we performed large box spin wave simulations using *SpinW*.^[^ ^22^ ^]^ We generated a lattice 12 unit‐cells in extent along the *b*‐axis with periodic boundary conditions along *a* and *c*. We then performed LSWT calculations with and without periodic boundary conditions along *b* using the Hamiltonian in Table 1, with and without *D*_1_ (DM on the first neighbor) exchange. The results are plotted in **Figure** **7**. The surface modes are clearly visible as the modes at lower energies than the bulk dispersions, and which disappear when periodic boundary conditions are applied.

Without DM interaction, the surface modes have a sinusoidal character, with the same dispersion for both surfaces. However, with a nonzero DM interaction, the modes split and shift left and right in reciprocal space, leading to crossing points at *h* = 0 and *h* = ±1/2 where the surface magnon modes have opposite group velocities. This signals a potentially chiral surface mode which can be induced in CrSBr. If a magnon mode is excited in the frequency and momentum window of a crossing point, its direction will be constrained by the dispersion to travel along a particular edge direction. The chiral edge modes may be visible in a thermal Hall experiment. Inducing these chiral edge modes via proximity effects is a real possibility: CrSBr heterostructures are already being fabricated^[^ ^12^ ^]^ and furthermore layered WTe_2_/Fe_3_GeTe_2_ were able to achieve 1.0 mJ m^−2^ proximity induced DM exchange,^[^ ^35^ ^]^ which would be 1.9 meV per Cr ion in CrSBr—larger even than our DM simulations in Figure 7.

As an aside, these simulations show that the DM interaction would also shift the mode energy minima from *Q* = 0 to an incommensurate value along *a*. This indicates that *D*_1_ would produce an incommensurate spiral spin modulation along *a* (the in‐plane direction perpendicular to the ordered moment).

## 4 Conclusion

In conclusion, we have measured the magnetic diffraction of CrSBr and confirmed the 2D *XY* A‐type antiferromagnetism. We also measured inelastic spin wave spectra of CrSBr and fitted the observed magnon modes to a linear spin wave model. We find a minimal magnetic exchange model with seven in‐plane exchanges accurately reproduces the experimental spectra, with both single‐ion and exchange anisotropy being too small to resolve. We also find no visible dispersion in the out of plane direction, confirming the highly 2D nature of CrSBr. We anticipate this experimentally derived Hamiltonian to be useful for calculating the behavior of this material in heterostructures and spintronic devices.

We then use this calculated spin wave model to predict the presence of a chiral edge mode if the nearest neighbor DM exchange interaction could be enhanced by proximity effects. These results suggest potential topological edge modes in CrSBr heterostructures is a future direction worth exploring.

## 5 Experimental Section

### Sample Synthesis

The following reagents were used as received unless otherwise stated: chromium powder (99.94%, −200 mesh, Alfa Aesar), sulfur pieces (99.9995%, Alfa Aesar), bromine (99.99%, Aldrich), and chromium dichloride, (anhydrous, 99.9%, Strem Chemicals).

For a starting material, high quality CrBr_3_ was synthesized from the elements (Cr: 1.78 g, 34.2 mmol and Br_2_: 8.41 g, 52.6 mmol) with one end of the tube maintained at 1000^~^°C and the other side at  50 °C with a water bath. Details of the reaction can be found in ref. [16]. *Caution: One end of the tube must be maintained below 120~*°C to prevent the tube from exploding from bromine overpressure.

A modified procedure from ref. [16] was used to synthesize large single crystals of CrSBr. Chromium (0.174 g, 3.35 mmol), sulfur (0.196 g, 6.11 mmol), and CrBr_3_ (0.803 g, 2.75 mmol) were loaded into a 12.7 mm O.D., 10.5 mm I.D. fused silica tube. The tube was evacuated to a pressure of ≈30 mtorr and flame sealed to a length of 20 cm. The tube was placed into a computer‐controlled, two‐zone, tube furnace. The source side was heated to 850~°C in 24 h, allowed to soak for 24 h, heated to 950~°C in 12 h, allowed to soak for 48 h, and then cooled to ambient temperature in 6 h. The sink side was heated to 950~°C in 24 h, allowed to soak for 24 h, heated to 850~°C in 12 h, allowed to soak for 48 h, and then cooled to ambient temperature in 6 h. The crystals were cleaned by soaking in a 1 mg mL^−1^ of CrCl_2_ aqueous solution for 1 h at ambient temperature. After soaking, the solution was decanted and the crystals were thoroughly rinsed with DI water and acetone. Residual sulfur residue was removed by washing with warm toluene.

### Neutron Experiments:

The neutron diffraction of CrSBr was measured with the TOPAZ diffractometer^[^ ^36^ ^]^ at Oak Ridge National Laboratory's SNS. TOPAZ used the neutron wavelength‐resolved Laue technique for data collection to measure a 3D volume from a stationary single‐crystal sample. Diffraction study was made on a plate‐shaped single crystal with dimensions 5 × 2.5 × 0.8 mm, oriented with the *a*‐axis vertical. Sample temperature was controlled by a Cryomech P415 pulse tube cryocooler. Data were collected using crystal orientations optimized with the CrystalPlan software in the range −161° to 180°^[^ ^37^ ^]^ at 200, 80, and 5 K. An order parameter curve heating from 5 to 200 K at a fixed rotation angle was also measured. As explained in detail in the Supporting Information, the BasIreps^[^ ^38^ ^]^ and JANA software packages^[^ ^39^ ^]^were used to perform a refinement to the magnetic Bragg intensities and a static ordered moment of 3.56(2) μ_B_ at *T* = 5 K was found. This is consistent with the theoretical static moment of a *S* = 3/2 Cr^3 +^ ion: *g*(3/2) = 3 μ_B_ plus a small orbital contribution.

The inelastic neutron spectrum of CrSBr was measured using the SEQUOIA spectrometer^[^ ^40^, ^41^ ^]^ at Oak Ridge National Laboratory's SNS.^[^ ^42^ ^]^ The sample consisted of 13 coaligned crystals with a total mass of 300 mg, aligned with the *c*‐axis vertical and glued to an aluminum plate using CYTOP glue^[^ ^43^ ^]^ (a picture is shown in the Supporting Information). The sample was mounted in a closed cycle refrigerator and cooled to a base temperature of 5 K. The scattering with incident energies *E*_*i*_ = 70 meV and *E*_*i*_ = 20 meV were measured.

For the SEQUOIA neutron measurements, the *T*0 chopper was set at 60 Hz, and high flux Fermi 1 chopper at 240 Hz was used, for *E*_*i*_ = 70 meV, and the neutron absorbing slits in front of the sample were set to provide the beam size 44 mm wide and 6 mm tall. The spectra was also measured with *E*_*i*_ = 20 meV neutrons using high resolution Fermi 2 chopper at 240 Hz, *T*0 chopper at 60 Hz. For the *E*_*i*_ = 70 meV data the sample was rotated a full 180° in 1° steps, but for the *E*_*i*_ = 20 meV data only 35° was rotated to capture the bottom of the dispersion around (1,1,0).

## Conflict of Interest

The authors declare no conflict of interest.

## Supporting information

Supporting Information

## Data Availability

Spin wave data is available for download at doi.org/10.13139/ORNLNCCS/1869252.
