# Supplementary material for: Spin Waves and Magnetic Exchange Hamiltonian in CrSBr
Source: Adv Sci (Weinh). 2022 Jul 7;9(25):2202467. doi: 10.1002/advs.202202467 (PMC9443475; doi:10.1002/advs.202202467)
Supplement: Supplementary file 1 — Supporting Information [file ADVS-9-2202467-s001.pdf]

ADVANCED SCIENCE
Open Access

Supporting Information

for *Adv. Sci.*, DOI 10.1002/advs.202202467

Spin Waves and Magnetic Exchange Hamiltonian in CrSBr

*Allen Scheie*, Michael Ziebel, Daniel G. Chica, Youn June Bae, Xiaoping Wang, Alexander I. Kolesnikov, Xiaoyang Zhu and Xavier Roy*

# Supplemental Information for Spin waves and magnetic exchange Hamiltonian in CrSBr

A. Scheie,[1] M. Ziebel,[2] D. G. Chica,[2] Y. J. Bae,[2] Xiaoping Wang,[1] A. I. Kolesnikov,[1] Xiaoyang Zhu,[2] and X. Roy[2]

[1]*Neutron Scattering Division, Oak Ridge National Laboratory, Oak Ridge, Tennessee 37831, USA*
[2]*Department of Chemistry, Columbia University, New York, NY 10027, USA*

This document shows additional details of the diffraction and refinement of CrSBr scattering, details of the spin wave fits, and plots of measured CrSBr neutron scattering from SEQUOIA alongside the fitted linear spin wave model.

## I. DIFFRACTION AND REFINEMENT

To analyze and fit the neutron diffraction peaks to a magnetic structure model, we decomposed the CrSBr space group into irreducible representations using the BasIreps package of the FullProf software suite [1]. The CrSBr *Pmmn* space group decomposes into six irreducible representations: $\Gamma_1$ - $\Gamma_6$, shown on the right hand side of Figure 1. Each of these six irreducible representations gives a unique signal in the magnetic diffraction pattern. Susceptibility and neutron order parameter curves indicate a second order (continuous) phase transition, which means the transition involves a single irreducible representation.

These calculated patterns are compared with the measured CrSBr scattering at 80 K and 5 K in Fig 1. Although the Bragg peaks are asymmetric and broadened due to sample imperfections, the magnetic order is nonetheless clear: the magnetic diffraction matches $\Gamma_4$, in-plane ferromagnetic order along $a$, alternating between layers to form an antiferromagnetic layered structure.

Because no discontinuity is visible in the $(0,1,\frac{3}{2})$ order parameter curve (see main text), we believe there is no additional low-temperature transition in the magnetic order at 30 K. Nevertheless, we can use the 5 K data to constrain the possible magnetic order at lower temperatures, allowing for an additional irreducible representation which would modify the magnetic structure. Fitting combinations of irreducible representations to the observed intensities, we find that the largest possible spin canting angle at 5 K is $14^\circ$ to within one standard deviation uncertainty, coming from the addition of $\Gamma_1$. However, the largest possible secondary irreducible representation weight is from the addition of $\Gamma_3$ which modulates the size of the ordered moments, up to $\pm 45\%$. Thus even were a low temperature transition to exist, its effect on the overall spin structure would be quite mild. However, both of these possibilities would involve a discontinuity in the $(0,1,\frac{3}{2})$ Bragg peak, which we do not observe.

## II. KOSTERLITZ-THOULESS PHYSICS

In the main text we show that the magnetic order parameter follows the 2D $XY$ critical exponent associated with Kosterlitz-Thouless (K-T) physics [2], indicating the possible existence of magnetic vortices in the paramagnetic phase. As shown in Figure 2, this result is robust against background subtracted fits.

One of the original predictions of Kosterlitz [3, 4] was that the high temperature susceptibility of a K-T system follows a universal curve

$$\chi(T) = A \exp(B\, t^{-1/2}) \tag{1}$$

where $A$ and $B$ are fitted constants and $t = (T - T_{KT})/T_{KT}$. Here $T_{KT}$ is the vortex binding transition, below which no free vortices can exist in the lattice. Fitting the high temperature CrSBr susceptibility to this formula, we obtain very good agreement down to $\sim$ 150 K as shown in Figure 3, with a fitted $T_{KT} = 106.3(5)$ K in the $b$ and $c$ directions and $T_{KT} = 93.7(3)$ K in the $a$ direction. This confirms that CrSBr behaves as a 2D K-T system, and potentially that around 100 K magnetic vortices and antivortices would bind together and annihilate. Intriguingly, the fitted $T_{KT}$ transition is nearly the same as the $T_S$ temperature identified by muon spin relaxation [5]. Whether this is coincidence or indicates a deep connection is a question left for future study.

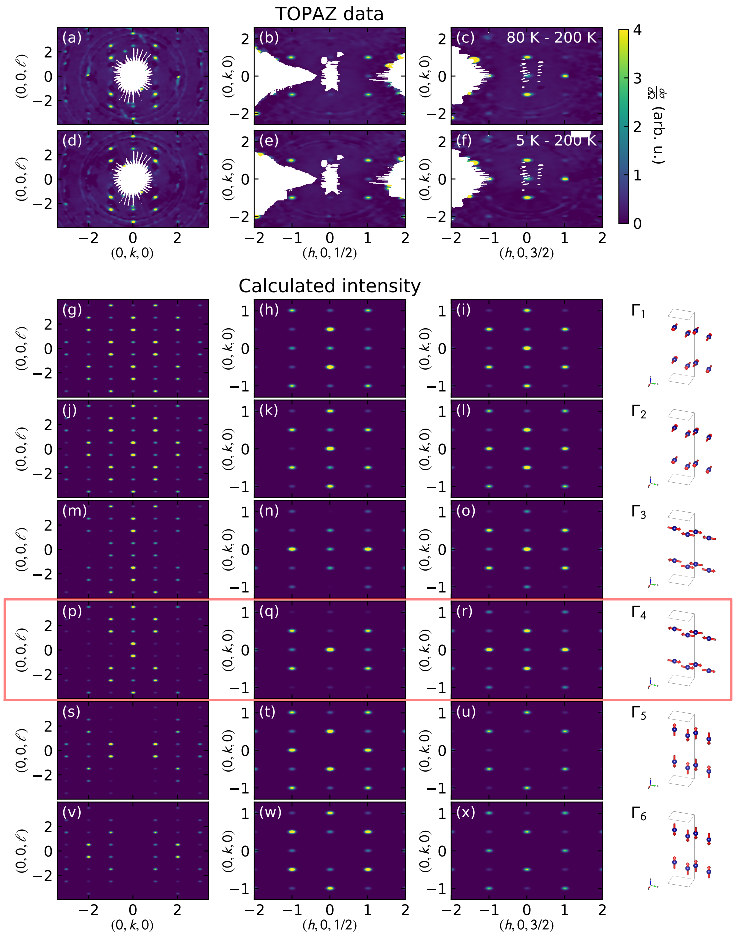


Figure 1. CrSBr single crystal diffraction and refinement. The top two rows show the experimental diffraction data, in the $(0, k, \ell)$ (left column), $(h, k, 1/2)$ (middle column), $(h, k, 3/2)$ (right column) scattering planes. The top row (a)-(c) shows the 80 K scattering with 200 K subtracted as background, and the second row (d)-(f) shows the same for 5 K scattering. Note that no new Bragg peaks appear at the lower temperatures. The bottom section (g)-(x) shows the calculated scattering intensity for the six possible irreducible representations for CrSBr, one for each row, with the magnetic structures shown on the right. The only structure that matches the observed scattering pattern is $\Gamma_4$.

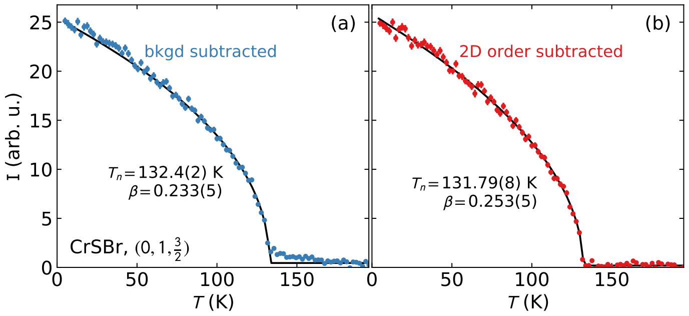


Figure 2. CrSBr order magnetic parameter measured on the $(0, 1, 3/2)$ Bragg peak (main text Figure 2), with the background (a) and with 2D order parameter (b) subtracted. The fitted critical exponent with the background subtracted data agrees with the main text $\beta = 0.231(6)$ to within uncertainty, but the fitted exponent with 2D order subtracted is noticeably higher. Intuitively, this makes sense: upon subtracting the 2D correlations, the result is more three-dimensional.

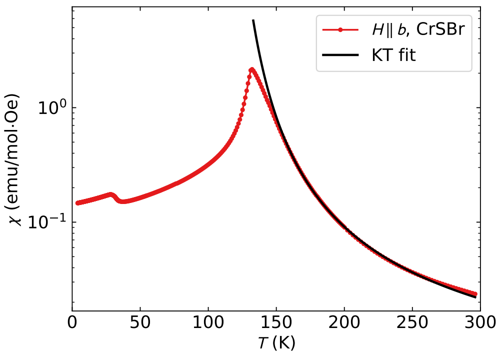


Figure 3. CrSBr magnetic susceptibility along $b$, from Ref. [6], fitted to Eq. 1 above the magnetic ordering transition. Between $\sim$ 150 K and 300 K, the susceptibility follows the K-T functional form.

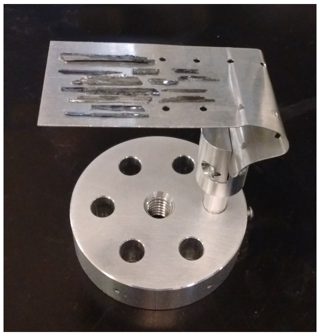

Figure 4. CrSBr sample used for the neutron experiment, consisting of 13 coaligned crystals glued to an aluminum plate for a total mass of 300 mg. The $c$ axis is vertical, and the longest crystal edge is along the $a$ axis.

## III. SEQUOIA EXPERIMENT DETAILS

The sample for this measurement is shown in Figure 4. It consists of 13 crystals coaligned with the $c$ axis vertical, glued to an aluminum plate with CYTOP glue [7].

To estimate background for this experiment, we moved the absorbing slits up by 9 mm so that the neutron beam illuminates the middle of the aluminum sample mount and not the CrSBr crystals. This was subtracted from the data as shown in Figure 5. The diffuse background is not eliminated, but it is reduced to the point where it is clear what the magnon modes are.

To increase the statistical clarity of the CrSBr scattering data, we symmetrized the data by folding it over high-symmetry directions as shown in Figure 6. The magnon modes are clear even without symmetrizing,

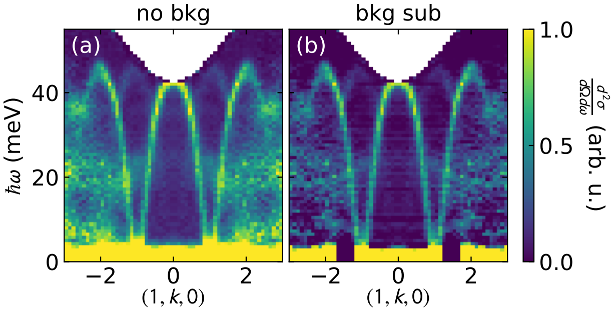


Figure 5. CrSBr scattering with and without the background subtracted. The background significantly reduces the intensity of the diffuse features whilst leaving the magnon intensity unchanged.

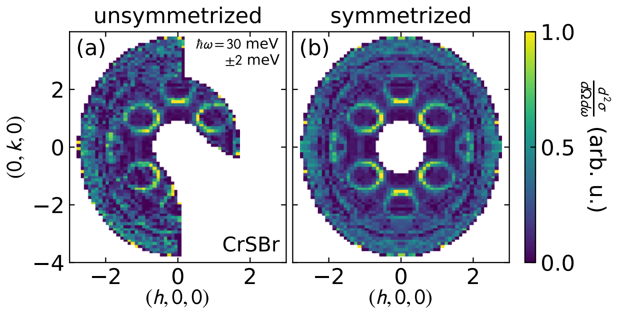


Figure 6. Symmetrized and unsymmetrized CrSBr data, showing a constant energy slice at $\hbar\omega = (30 \pm 2)$ meV.

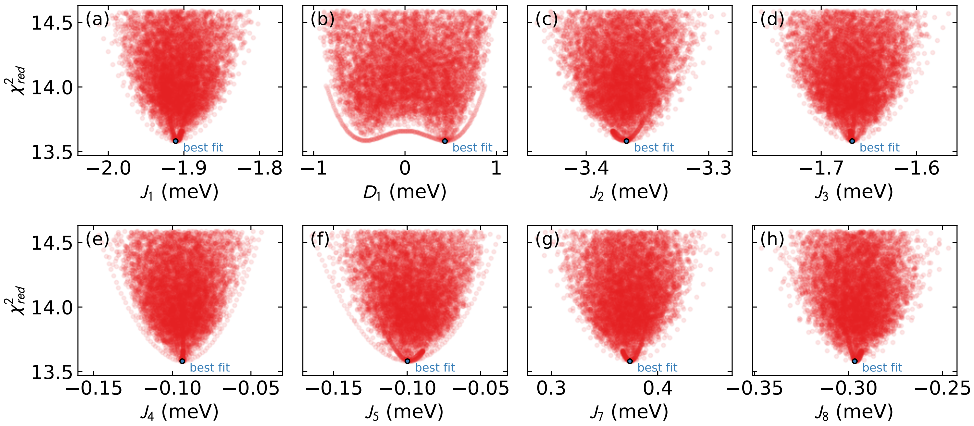


Figure 7. Possible Hamiltonian solutions within $\Delta\chi^2_{red} = 1$ of the global optimum fit (see text). Each panel shows the range of such solutions, which we take as an estimate of uncertainty. The small blue circle represents the best fit values. Note that the DM interaction in panel (b) is symmetric about $D_1 = 0$.

but applying the symmetry operations increases the effective signal intensity.

## IV. SPIN WAVE FITS

As noted in the main text, the spin wave Hamiltonian was fitted to the energies of magnon modes extracted from the scattering data. This optimization was repeated several times using different starting parameters, and always converged to the same solution.

Uncertainty was estimated by calculating the $\Delta\chi^2_{red} = 1$ contour about the best fit Hamiltonian. The extremities of this contour along each parameter is then a measure of the one standard deviation uncertainty [8]. We estimate the $\Delta\chi^2_{red} = 1$ using the same method as employed in Ref. [9], by generating a family of solutions within $\Delta\chi^2_{red} = 1$ of the best fit Hamiltonian. We first systematically vary a single parameter, slowly increasing or decreasing its value while re-fitting all other values, keeping solutions within $\Delta\chi^2_{red} \leq 1$. Then, using principal component analysis to define the vectors along parameter space to search, we perform a random Monte Carlo search to generate additional solutions within $\Delta\chi^2_{red} = 1$. The ranges of valid solutions are plotted in Figure 7, which are used to define the statistical uncertainty in main text Table I.

This method of calculating uncertainty also yields the correlations between the various fitted parameters. Figure 8 shows the correlations between different parameters on $J_2$ in the best fit family of solutions. This is then used to calculate a correlation matrix, as shown in Figure 9.

The case of the DM exchange $D_1$ is somewhat unique as it effects only some of the dispersion $Q$ points. (It leaves integer and half-integer $h$ unchanged for instance.) So for estimating the uncertainty of $D_1$, we modified $\chi^2_{red}$ to be $\chi^2$ per data point for which DM has a noticeable effect. This reduced the uncertainty from $\pm 0.5$ meV to $\pm 0.4$ meV. But in either case, the uncertainty in $D_1$ overlaps with zero.

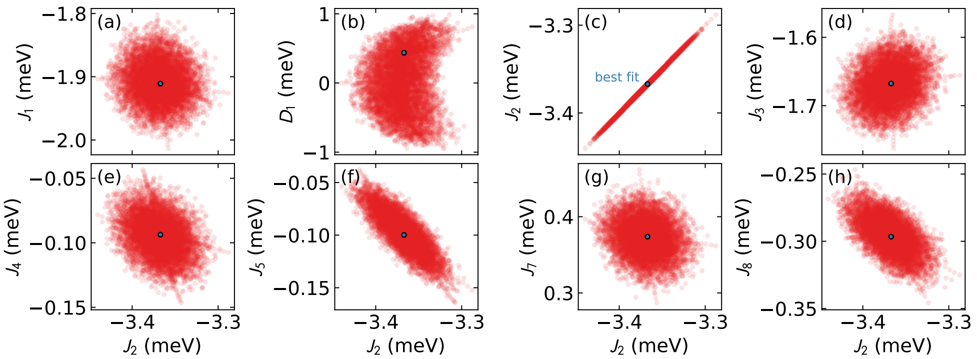


Figure 8. Hamiltonian solutions within $\Delta\chi^2_{red} = 1$ plotted against $J_2$, as an example of correlations between fitted parameters. A circular distribution shows no correlation, but a tilted ellipsoidal distribution shows nonzero correlation. The small blue circle represents the best fit values.

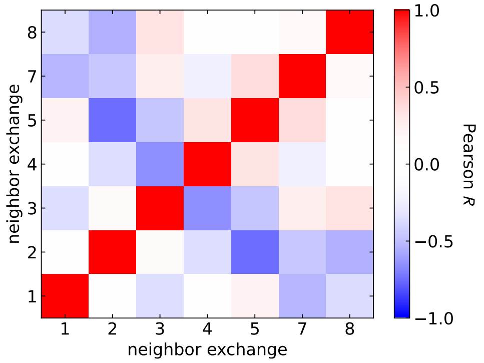


Figure 9. Correlation matrix for nonzero $J_n$ from the CrSBr spin wave mode fits, generated from the data in Figure 8 via a Pearson $R$ calculation. Red indicates positive correlation, blue indicates negative correlation.

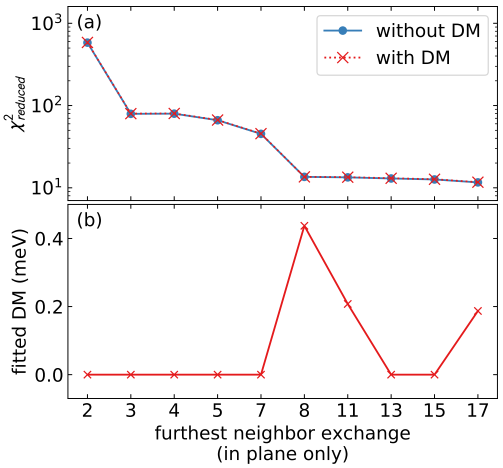


Figure 10. (a) dependence of the best fit $\chi^2_{\text{red}}$ on the number of neighbors $n$, both with and without a DM term included. The overall $\chi^2$ is barely different when $D_1$ is included, and in fact is slightly higher for all $n$. (b) Best fit $D_1$ value plotted against $n$, showing the fitted value is unstable with $n$. Thus the DM cannot be accurately determined by this data, but is small enough that it makes no difference to fit.

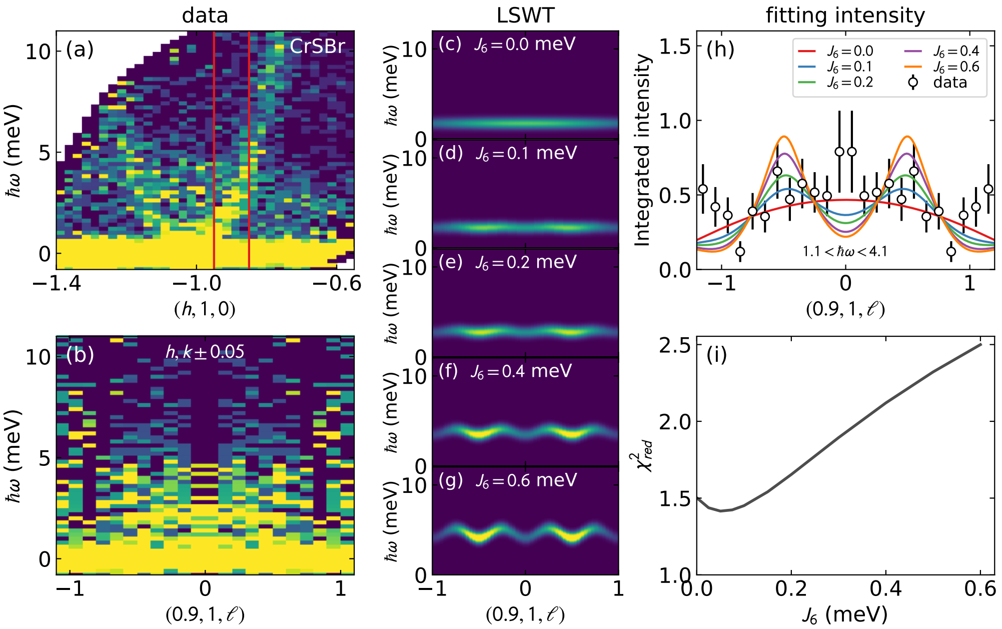


Figure 11. Fitted dispersion in the out of plane ($\ell$) direction. (a) shows $E_i = 20$ meV data, with the red lines indicating the integrated region in panel (b). Panels (c) through (g) show linear spin wave simulations of the CrSBr dispersion with varying values of the inter-plane exchange $J_6$. As the interplane exchange grows larger, the intensity concentrates around $\ell = \pm 1/2$. Panel (h) shows the integrated intensity from panel (b) in the window between 1.1 meV and 4.1 meV compared to the linear spin wave simulations. Panel (i) shows the reduced $\chi^2$ as a function of $J_6$, giving a best fit of $J_6 = 0.05$ meV, with an uncertainty up to 0.5 meV.

Furthermore, although with $n = 8$ neighbors included in the fit (what is plotted in Figure 7) gives a best fit value of $D_1 \approx 0.4$, this value is unstable with $n$. As shown in Figure 10, the best fit $D_1$ value varies unpredictably as the number of fitted $J_n$ increases—and in most cases refines to zero. Additionally, the overall $\chi^2_{red}$ is never improved by adding a $D_1$ term to the Hamiltonian, as shown in Figure 10(a). Because of this, we cannot say that $D_1$ is nonzero in CrSBr, but based its statistical uncertainty it could be as large as 0.8 meV.

## V. INTER-PLANE EXCHANGE

As noted in the main text, no inter-plane magnetic dispersion is detectable to within the resolution of the neutron experiment. Nevertheless, we can attempt to put an upper bound on the $J_6$ inter-plane exchange by examining the lowest energy inelastic scattering, as shown in Figure 11.

As is shown by the LSWT calculations in Figure 11, small nonzero $J_6$ causes the low energy magnon spectral weight to concentrate around $\ell = \pm 1/2$. Experimentally, no such intensity concentration is observed. Fitting the $\ell$ dependence of the intensity at $h = 0.9$, $k = 1.0$ (displaced from the $(1, 1, \ell)$ so that the inelastic magnetic signal is distinguishable from the elastic background) in Figure 11(h), we find that the best fit $J_6$ is 0.05 meV with an uncertainty of 0.5 meV (estimated from a one standard deviation in reduced $\chi^2$). The error bars are unfortunately too large to constrain the fit very well, but it is clear that $J_6$ is quite small in CrSBr, and the system acts very two-dimensionally.

## VI. MAGNETIC ANISOTROPY

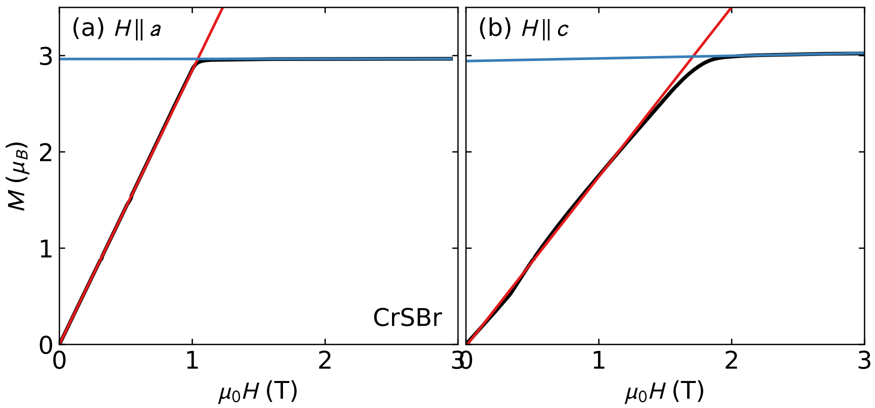


Figure 12. CrSBr 2 K magnetization as a function of field in the (a) $a$ direction and (b) $c$ direction. The black curve indicates the experimental data, the red and blue lines are linear fits.

Although the neutron experiments are unable to resolve the low energy magnon gap, we can estimate the magnetic anisotropy using isothermal magnetization. For magnetocrystalline anisotropy calculations, a single crystal was oriented along the $a$ or $c$ axis and attached to a quartz paddle with GE varnish, and field-dependent magnetization measurements were collected at 2 K using the vibrating sample magnetometry module of a Quantum Design PPMS Dynacool system. The same crystal was used for both $a$- and $c$-axis measurements. The magnetocrystalline anisotropy (defined here as the energy difference between the $b$-axis-magnetized state and the $a$- or $c$-axis magnetized state) was calculated using the Stoner-Wohlfarth model [10]. By measuring the magnetization as a function of field at 2 K, we estimate the saturation magnetic field $H_{sat}$ and magnetization $M_{sat}$ by linearly fitting the low field and high field magnetization and finding the intersection as shown in Figure 12. By calculating the effective anisotropy parameter $K_{eff} = (\mu_0 H_{sat} M_{sat})/2$ for each direction as in Refs. [11, 12], we can find the difference between the anisotropy constants for the $a$ or $c$ axis and the $b$-axis. These are 144 $\mu$eV/Cr ($c$-axis compared to $b$) and 90 $\mu$eV/Cr ($a$-axis compared to $b$). These estimates are remarkably close to the measured magnon gaps from transient reflectance spectroscopy of 0.141(4) meV and 0.102(3) meV [13]. Taken as estimates of the spin wave gap, these are much to small to be resolved with the reported neutron data. Thus, on short timescales (high frequencies), CrSBr can be approximated as a very isotropic 2D magnet.

We note that the values calculated here do not account for possible effects of shape anisotropy, which would be largest for the $c$-axis, given the plate-like morphology of the crystals. However, correcting the magnetization data with a demagnetization factor near unity ($N = 0.9$) alters the calculated anisotropy energy for the $c$-axis by less than 10%. As such, possible effects of shape anisotropy do not alter any conclusions discussed herein.

## VII. DISPERSION PLOTS

Figures 13 through 26 show the measured dispersion compared to the linear spin wave theory (LSWT) simulated intensity from the best fit Hamiltonian in the main text. Note that the data were integrated over a finite region in $\ell$, rendering the optic modes faintly visible whereas at $\ell = 0$ they would have zero intensity. To account for this, the LSWT simulations were carried out for $\ell = 1/2$. The bin width in $h$ and $k$ was $\pm 0.1$ reciprocal lattice units, and the linear spin wave simulations assumed a Gaussian in energy with a constant width of 4 meV (chosen to visually match the experimental plots). The third panel on the right shows line plots of the calculated dispersion curves. If experimental data were extracted from the particular slice and used to constrain the fit, they are plotted overtop the calculated dispersion. In some cases, the data were too noisy to extract the modes, but one can see that the calculated magnon intensity matches the features in the data. All inelastic neutron scattering data and spin wave analysis code is available at doi.org/10.13139/ORNLNCCS/1869252.

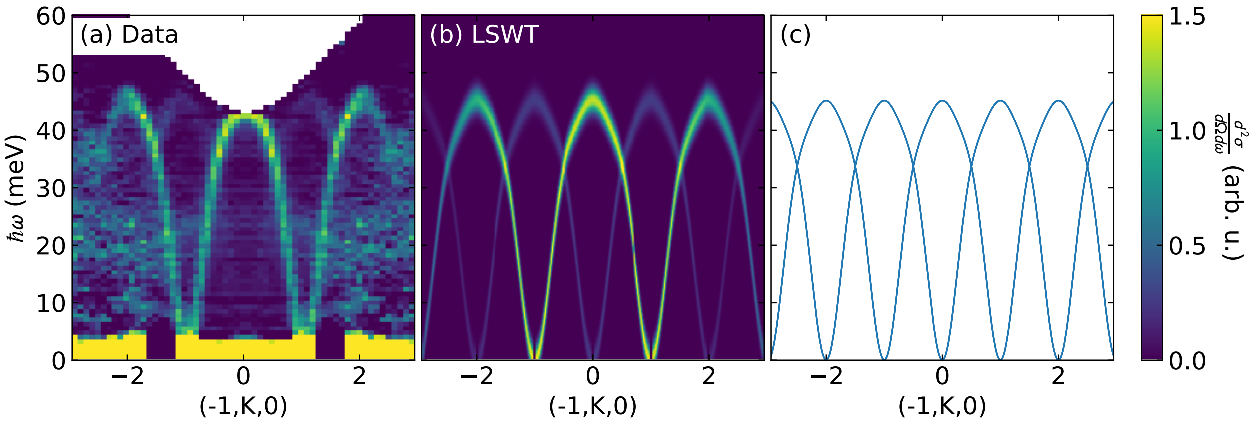


Figure 13. Experimental CrSBr scattering compared to LSWT fit.

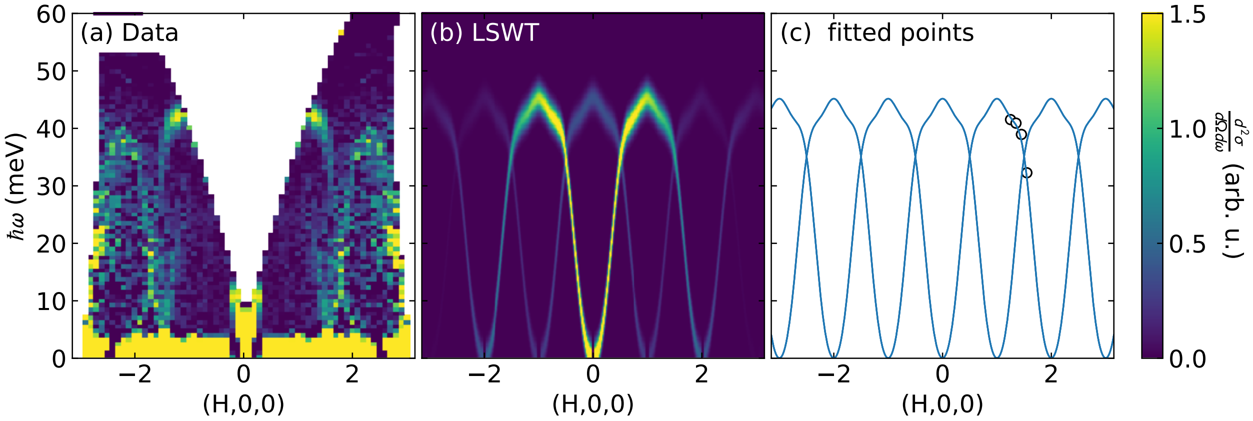


Figure 14. Experimental CrSBr scattering compared to LSWT fit.


[1] J. Rodrguez-Carvajal, Recent advances in magnetic structure determination by neutron powder diffraction, Physica B: Condensed Matter **192**, 55 (1993).

[2] S. T. Bramwell and P. C. W. Holdsworth, Magnetization and universal sub-critical behaviour in two-dimensional XY magnets, Journal of Physics: Condensed Matter **5**, L53 (1993).

[3] J. M. Kosterlitz, The critical properties of the two-dimensional xy model, Journal of Physics C: Solid State Physics **7**, 1046 (1974).

[4] C. A. Cornelius, P. Day, P. J. Fyne, M. T. Hutchings, and P. J. Walker, Temperature and field dependence of the magnetisation of $Rb_2CrCl_4$: a two-dimensional easy-plane ionic ferromagnet, Journal of Physics C: Solid State Physics **19**, 909 (1986).

[5] S. A. López-Paz, Z. Guguchia, V. Y. Pomjakushin, C. Witteveen, A. Cervellino, H. Luetkens, N. Casati, A. F. Morpurgo, and F. O. von Rohr, Dynamic magnetic crossover at the origin of the hidden-order in van der waals antiferromagnet CrSBr, arXiv preprint arXiv:2203.11785 (2022).

[6] E. J. Telford, A. H. Dismukes, K. Lee, M. Cheng, A. Wieteska, A. K. Bartholomew, Y.-S. Chen, X. Xu, A. N. Pasupathy, X. Zhu, *et al.*, Layered antiferromagnetism induces large negative magnetoresistance in the van der Waals semiconductor CrSBr, Advanced Materials **32**, 2003240 (2020).

[7] K. C. Rule, R. A. Mole, and D. Yu, Which glue to choose? A neutron scattering study of various adhesive materials and their effect on background scattering, Journal of Applied Crystallography **51**, 1766 (2018).

[8] W. H. Press, S. A. Teukolsky, W. T. Vetterling, and B. P. Flannery, *Numerical recipes 3rd edition: The art of scientific computing* (Cambridge university press, 2007).

[9] A. Scheie, P. Laurell, P. A. McClarty, G. E. Granroth, M. B. Stone, R. Moessner, and S. E. Nagler, Spin-exchange hamiltonian and topological degeneracies in elemental gadolinium, Phys. Rev. B **105**, 104402 (2022).

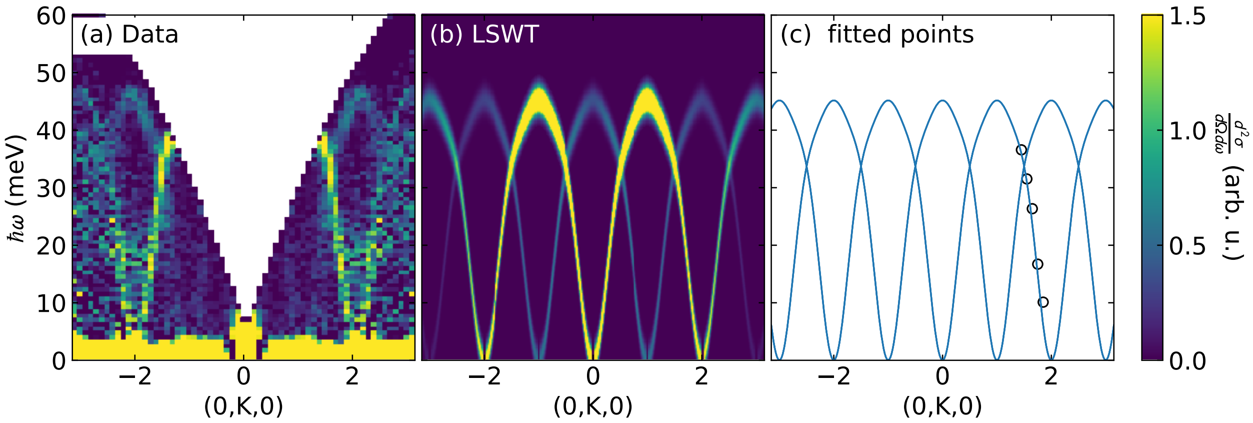


Figure 15. Experimental CrSBr scattering compared to LSWT fit.

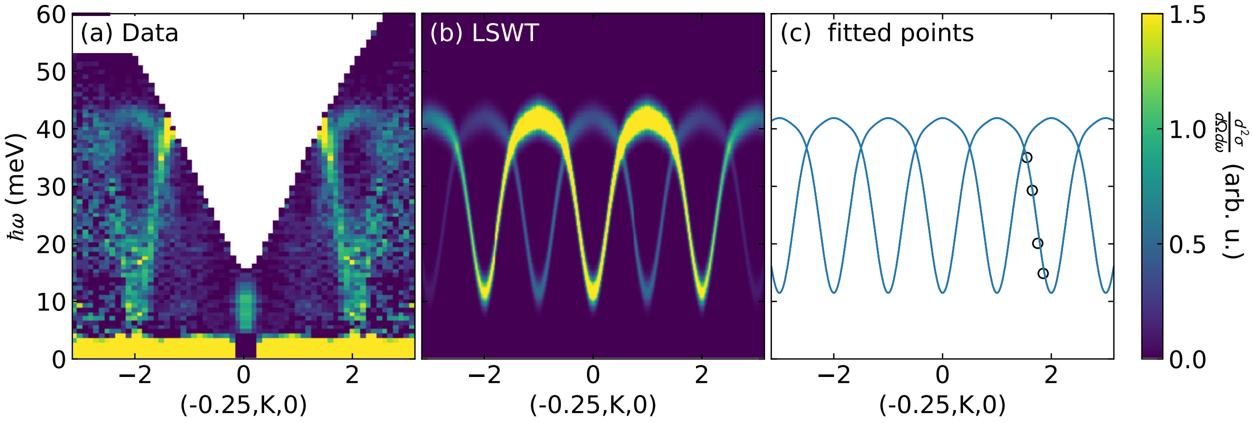


Figure 16. Experimental CrSBr scattering compared to LSWT fit.


[10] E. C. Stoner and E. Wohlfarth, A mechanism of magnetic hysteresis in heterogeneous alloys, Philosophical Transactions of the Royal Society of London. Series A, Mathematical and Physical Sciences **240**, 599 (1948).

[11] O. Zhdanova, M. Lyakhova, and Y. G. Pastushenkov, Magnetocrystalline anisotropy, magnetization curves, and domain structure of FeB single crystals, The Physics of Metals and Metallography **112**, 224 (2011).

[12] O. V. Zhdanova, M. B. Lyakhova, and Y. G. Pastushenkov, Magnetic properties and domain structure of FeB single crystals, Metal Science and Heat Treatment **55**, 68 (2013).

[13] Y. J. Bae, J. Wang, J. Xu, D. G. Chica, G. M. Diederich, J. Cenker, M. E. Ziebel, Y. Bai, H. Ren, C. R. Dean, *et al.*, Exciton-coupled coherent antiferromagnetic magnons in a 2d semiconductor, arXiv preprint arXiv:2201.13197 (2022).

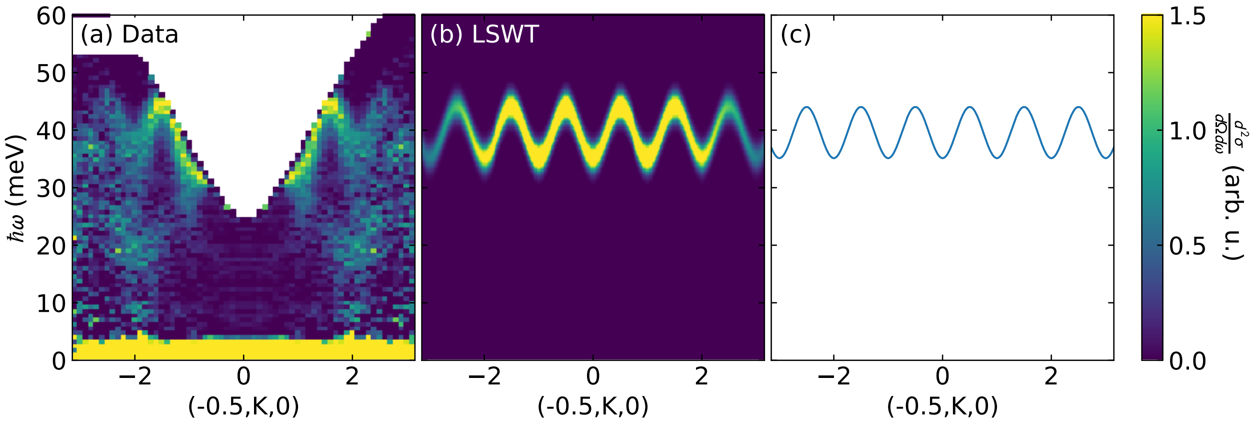


Figure 17. Experimental CrSBr scattering compared to LSWT fit.

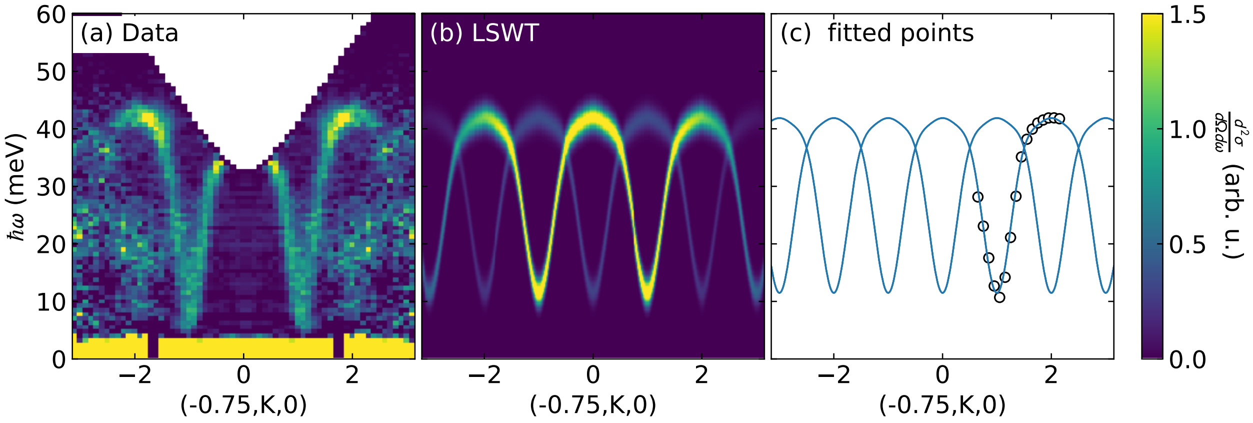


Figure 18. Experimental CrSBr scattering compared to LSWT fit.

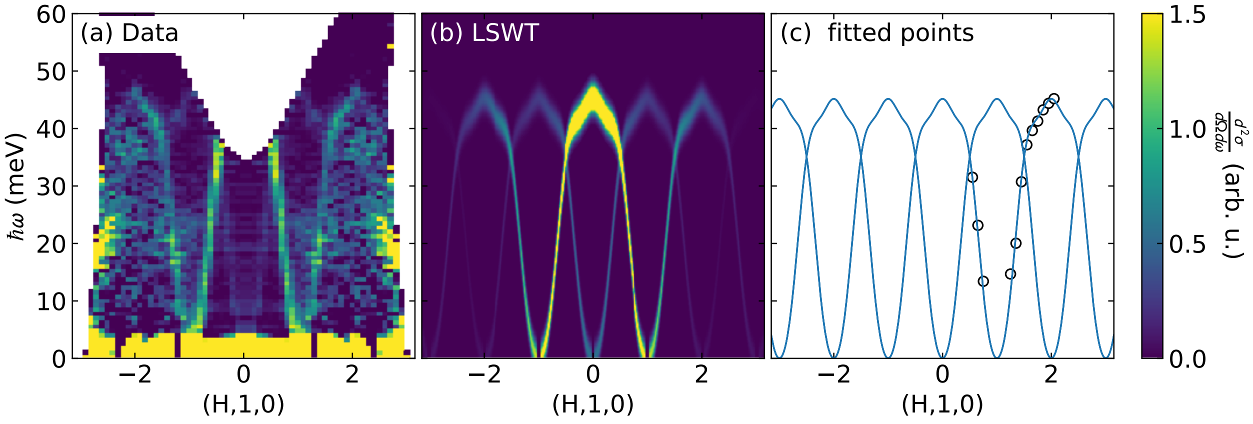


Figure 19. Experimental CrSBr scattering compared to LSWT fit.

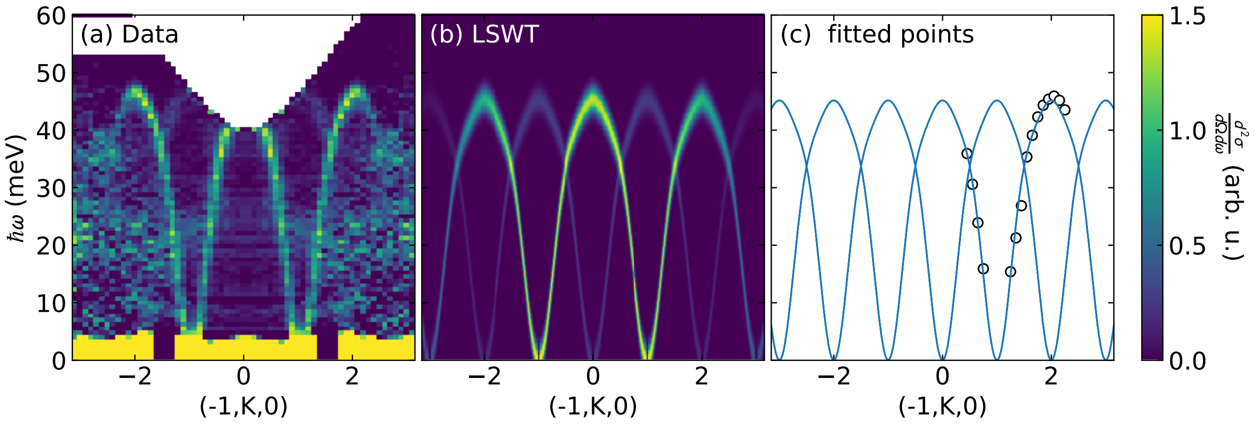


Figure 20. Experimental CrSBr scattering compared to LSWT fit.

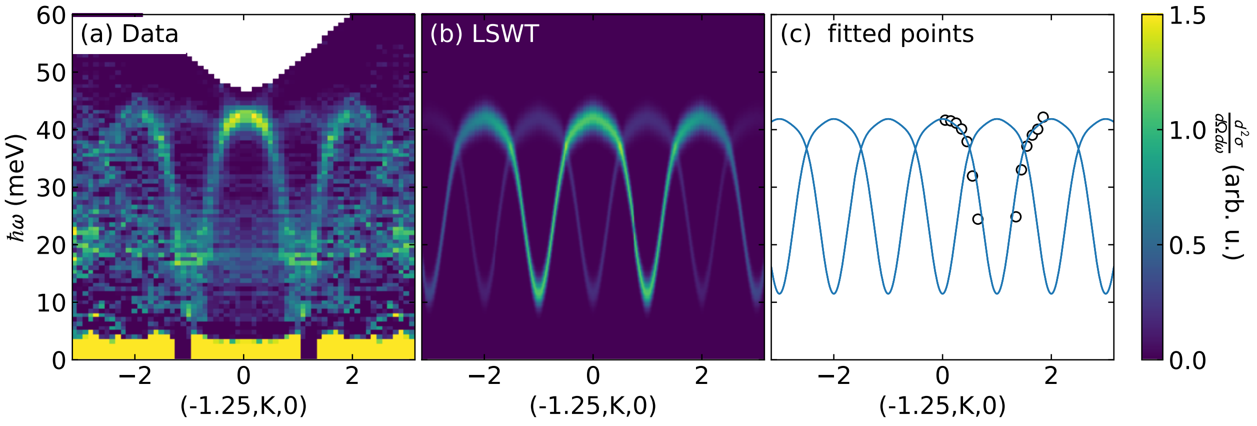


Figure 21. Experimental CrSBr scattering compared to LSWT fit.

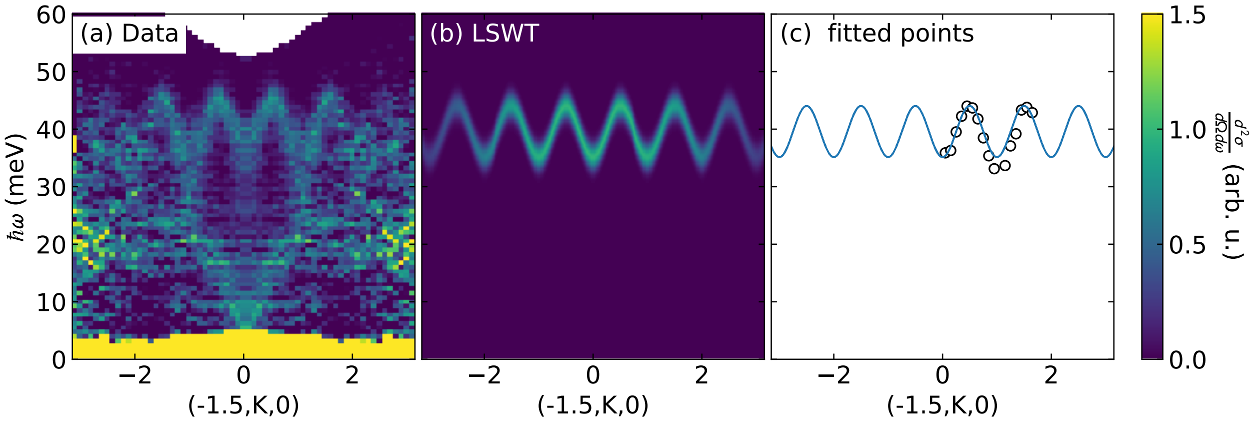


Figure 22. Experimental CrSBr scattering compared to LSWT fit.

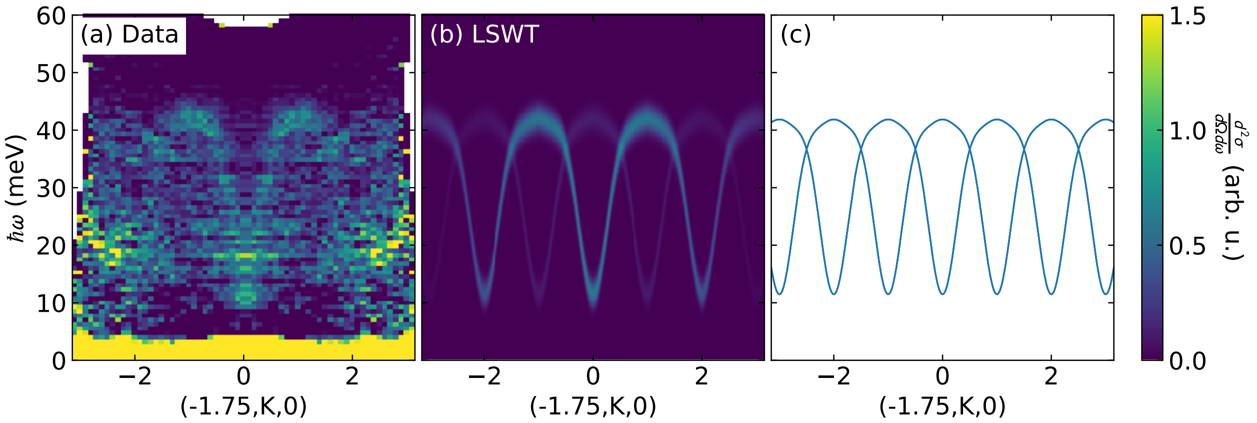


Figure 23. Experimental CrSBr scattering compared to LSWT fit.

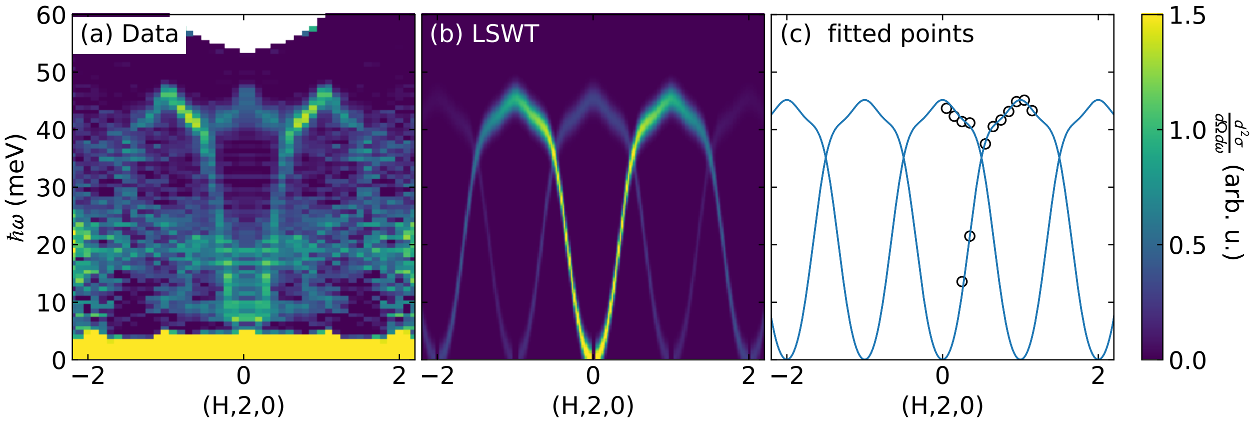


Figure 24. Experimental CrSBr scattering compared to LSWT fit.

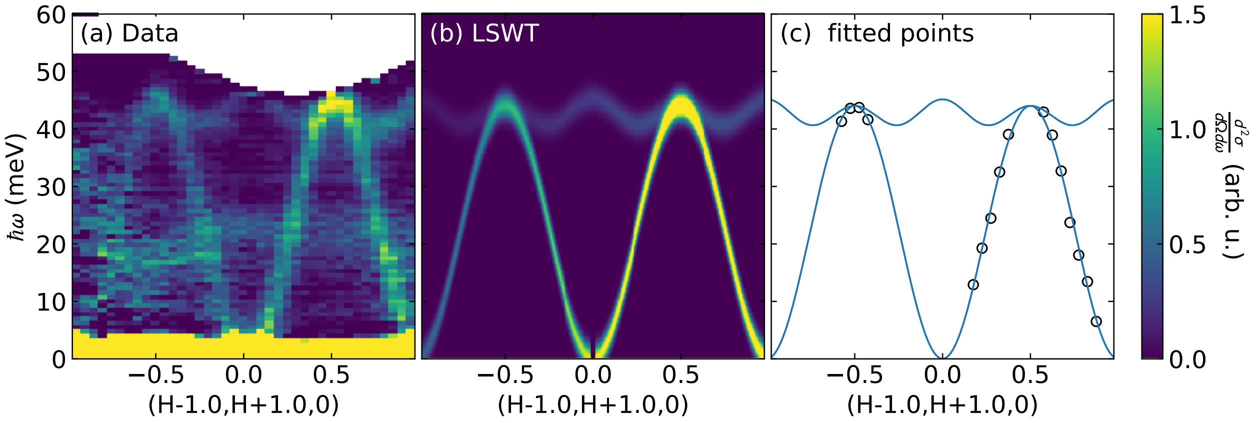


Figure 25. Experimental CrSBr scattering compared to LSWT fit.

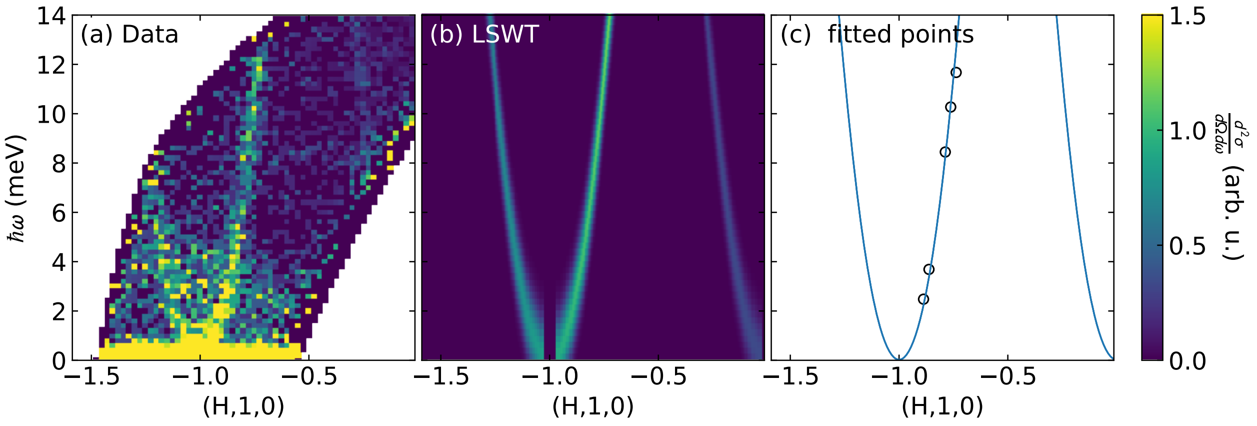


Figure 26. Experimental CrSBr scattering compared to LSWT fit.
